# Development, Implementation, and Evaluation of an e-Learning in Integrative Oncology for Physicians and Students Involving Experts and Learners: Experiences and Recommendations

**DOI:** 10.1007/s13187-022-02189-1

**Published:** 2022-07-01

**Authors:** Anita V. Thomae, Alizé A. Rogge, Stefanie M. Helmer, Katja Icke, Claudia M. Witt

**Affiliations:** 1 Institute for Complementary and Integrative Medicine, University Hospital Zurich and University of Zurich, Sonneggstrasse 6, 8091 Zurich, Switzerland; 2 Department of Psychosomatic Medicine, Center for Internal Medicine and Dermatology, Charité – Universitätsmedizin Berlin, Corporate Member of Freie Universität Berlin and Humboldt-Universität zu Berlin, 10117 Berlin, Germany; 3 Institute for Health and Nursing Science, Charité – Universitätsmedizin Berlin, Corporate Member of Freie Universität Berlin and Humboldt-Universität zu Berlin, 13353 Berlin, Germany; 4 Institute for Social Medicine, Epidemiology, and Health Economics, Charité – Universitätsmedizin Berlin, Corporate Member of Freie Universität Berlin and Humboldt-Universität zu Berlin, 10117 Berlin, Germany

**Keywords:** Integrative oncology, e-Learning, Stakeholder involvement, Blended learning, Cancer, Complementary medicine, Education, Training

## Abstract

In this project, an e-Learning program for complementary and integrative medicine in oncology was systematically developed, implemented, and evaluated in a stepwise procedure. Learning objectives and content were defined within the KOKON project network, considering the educational competencies for integrative oncology. To design a valuable e-Learning, experts were involved in all relevant steps of the process, as well as stakeholders from various target groups (undergraduates: medicine students, postgraduates: oncology physicians). We used mixed methods including quantitative surveys, progress tests, and qualitative focus groups. The developed e-Learning program led to a significant measurable knowledge gain about complementary and integrative medicine. In parallel, physicians and students were subjectively satisfied with the training. For the majority of e-Learning elements, the needs of both target groups are comparable. Furthermore, both groups emphasized the value of formative assessment tools for gaining knowledge. From the various surveys and experiences collected in this project, we derive recommendations for others developing e-Learning programs.

**Supplementary Information:**

The online version contains supplementary material available at 10.1007/s13187-022-02189-1.

## Introduction

Complementary and integrative medicine is a topic cancer patients are interested in and medical staff needs to be informed about. Patients desire reliable information and wish for a subsequent integration into their cancer therapy [1, 2].

Integrative oncology includes a patient-centered, evidence-informed approach to cancer care that utilizes mind and body practices, natural products, and/or lifestyle modifications from different traditions alongside conventional cancer treatments [3]. Health professionals seek reputable evidence-based information about complementary and integrative medicine [1, 4].

The advantages of e-Learning courses on the transmission of cognitive skills are known [5, 6]. In contrast to face-to-face teaching, no travel is necessary, the training can be integrated into the various daily schedules regardless of time and place, and learners can choose their own pace. By facilitating self-directed learning, selected content can be deepened depending on knowledge and interest [7]. Collaborative learning and exchange between learners can be supported on suitable platforms. Additionally, a wide range of multimedia tools can be integrated into the training. In blended learning programs, the respective benefits from e-Learning and face-to-face teaching are used at their best by combining both forms of teaching.

During the COVID-19 pandemic, the need for digital teaching offers increased rapidly [8]. Since students have to deal with a massive number of digital teaching offers, there is also a demand for higher-quality teaching programs.

KOKON (a German acronym for Competence Network for Complementary Medicine in Oncology) is a German joint research program aiming (among other things) to generate easily accessible information about complementary and integrative medicine for health care professionals [9]. Within this project, e-Learning courses for physicians have already been developed and evaluated in a previous study. The study of Blödt et al. with only small e-Learning parts found a distinct need for a more intensive training on the topic of complementary and integrative medicine. To better integrate such a training into very different daily routines, the usage of an e-Learning program was recommended as the most suitable format [10]. Hence, the development of an e-Learning program is part of the KOKON-KTO-framework in a subsequent funding period of the KOKON research program. This e-Learning as described here is combined with an onsite workshop (focusing on communication skills) as a blended learning training [11].

Based upon the e-Learning developed for oncology physicians in Germany, the e-Learning was revised for medical students in Switzerland and was included in the curriculum of medical students and combined with onsite workshops (focusing on practical aspects) as part of a blended learning training [12].

To date, there is no evaluated e-Learning program available that teaches post-order undergraduates the basics and procedures of integrative oncology. Although several e-Learning courses in teaching and higher education exist, to our knowledge, systematic development and evaluation have not been published. The aim of this project was to design, implement, and evaluate an e-Learning program on complementary and integrative medicine content and adapt the didactical formats to the needs of two different groups, either oncology physicians as postgraduates or medical students as undergraduates. The results are reported here and recommendations are derived for the development of other e-Learning programs.

## Methodological Approach

### Sequence of the Individual Project Phases

The e-Learning program was developed involving an expert panel and stakeholders in regard to the two target groups (postgraduate: oncology physicians and undergraduate: medical students). The project consisted of six phases (Supplementary Material S1), and the results of each phase were included in the further development in the subsequent phases. Because of this, some results that support a better understanding of the methodological approach were included in the “Methodological Approach” section. The other results are displayed in the “Results” section.

The final e-Learning program was implemented in Moodlerooms/Open LMS 2005.

The project was submitted to the respective ethics committee, which decided that the project needs no ethics approval (Cantonal Ethics Committee Zurich, BASEC-No Req-2017–00497).

#### Phase 1: Development of a Concept for e-Learning Program and Content Development

The learning objectives and topics of the e-Learning program were selected by various members of the KOKON network, taking into account complementary and integrative medicine topics commonly discussed with cancer patients [9, 11, 13]. Learning objectives followed Bloom’s taxonomy (Supplementary material S2), enabling a hierarchical order of the learning process [14]. The e-Learning program was developed in a format that could be integrated into a blended learning training to cover higher taxonomy levels.

An expert committee of the KOKON network provided advice during the entire development process of the e-Learning. Together with the e-Learning editor, this expert committee also decided on the structure for the e-Learning lessons. Formats such as texts/videos/graphics were selected taking into account the content difficulty and expected need for content adjustment.

Researchers and clinicians served as authors of e-Learning lessons and developed the content with the respective expertise supported by an e-Learning editor. The content of the lessons was tailored to the learning objectives according to constructive alignment rules [15]. Each manuscript and the corresponding assessment questions were reviewed by at least two members of the expert panel and revised by the authors if necessary. The authors had the final responsibility for the content.

#### Phase 2: Assessing Expectations and Wishes for e-Learning

A total of 20 participants from the two target groups of the e-Learning program were recruited as stakeholders.

- Undergraduate perspective: Students: Medicine master’s students of the University of Zurich (UZH), with basic complementary medicine knowledge gained in an elective course on complementary medicine; *N* = 14 and Head Students Affairs at the Dean’s Office of the Medical Faculty, UZH; *N* = 1
- Postgraduate perspective: Oncology physicians (clinics for oncology and gynecology): University Hospital Zurich and Lucerne Cantonal Hospital; *N* = 5

Selected lessons from the e-Learning program were implemented on the e-Learning platform. Before accessing the lessons, the expectations and wishes of the 20 participants regarding e-Learning were assessed quantitatively (online and paper pencil survey). Specified items were rated using a 10-point numeric rating scale (1 = strongly disagree to 10 = strongly agree).

After finishing the lessons, subjective satisfaction with interface and design, technical usability, navigation, and time requirements was analyzed. These latter results are not shown here but were included in the further development.

#### Phase 3: Importance of e-Learning Elements for Knowledge Gain

Considering the expectations assessed in phase 2, additional selected lessons of the e-Learning program were developed and implemented online.

After completing the e-Learning program, the following stakeholders judged the importance of various elements of the e-Learning program for their knowledge gain (online and paper pencil survey).

- Undergraduate perspective:
  - Medicine master’s students of the UZH, with basic complementary medicine knowledge from the elective course on complementary medicine; *N* = 4
  - Medicine master’s students of the UZH, currently enrolled in the elective course on complementary medicine; *N* = 12
  - Head Students Affairs at the Dean’s Teaching Office of the Medical Faculty, UZH; *N* = 1
- Postgraduate perspective:
  - Oncology physicians (internal medicine and gynecology): University Hospital Zurich and Lucerne Cantonal Hospital; *N* = 5

#### Phase 4: Knowledge Gain Assessed by a Progress Test

Taking into account the results from phases 2 and 3, the e-Learning program was implemented completely with 5 learning units of 45 min each, including a selection of multimedia elements. This e-Learning was integrated into the elective course from the UZH on complementary medicine as a part of a blended learning concept. The 15 medicine master’s students from the UZH enrolled in this course were selected as the test group.

For three different topics of the course (Chinese medicine, mind body medicine, whole medical systems), the knowledge gained by the e-Learning was tested using a progress test format, which was included in the time frame of 5 learning units [16]: The students initially answered a total of 23 single choice questions (with 4 to 6 answer options) from the various areas before and after completing the e-Learning program.

Single choice questions were designed following evidence-based rules for writing multiple choice questions [17]. The questions were aligned to the learning objectives and lesson content according to the principles of constructive alignment [15].

#### Phase 5: Qualitative Interviews with Students: Focus Group Interview

After completing the blended learning course in complementary medicine, the 15 students from phase 4 were interviewed in a focus group about their satisfaction with the e-Learning program and possibilities for improvement. Students had the possibility to express their opinions individually. Results were summarized based on their importance for the students.

#### Phase 6: Integration of e-Learning Program in the KOKON-KTO Training for Oncology Physicians

The e-Learning program was integrated into the KOKON-KTO training for oncology physicians. The KOKON-KTO training is a blended training and aims to enable oncology physicians to provide advice on complementary and integrative medicine topics in a semi-standardized conversation. Thirty-seven oncology physicians from Germany and Switzerland with little experience in complementary medicine completed the training [11, 18].

## Results

### Phase 1: Development of a Concept for e-Learning Program and Content Development

To map the complementary medicine topics in the e-Learning program specifically to the target group and to ensure the achievement of learning objectives at the same time, a multistage process was defined (Supplementary material S3).

First, the expert panel chose topics and learning objectives. Hereafter, the content and digital formats were determined together with the respective authors and the e-Learning editor. After reviewing the manuscript by the expert panel and integrating necessary adaptations in content and formats, the manuscript was finalized and implemented on the learning platform. The lessons were tested stepwise involving stakeholders. The results of these tests were integrated for further implementation on the learning platform before the finalization of the e-Learning program. The e-Learning editor was substantially involved in each step to ensure a seamless transition of the individual steps. To focus on the essentials of each topic and facilitate learners’ orientation despite a large number of topics, the following standard structure was defined for all lessons by the expert panel and the editorial staff: terminology, mechanisms and application, effectiveness and safety, conclusion, and bibliography. Topic-specific adjustments and extensions were possible if needed within single lessons.

### Phase 2: Assessing Expectations and Wishes for e-Learning

Expectations and wishes of stakeholder groups concerning (multimedia) elements and communication tools in online courses are shown in Table 1.

**Table 1:** Expectations and wishes for e-Learning as assessed before attending e-Learning program

	Total (*N* = 20) median/IQR	Undergraduate^1^ (*N* = 15) median/IQR	Postgraduates^2^ (*N* = 5) median/IQR
*From my point of view, the following multimedia elements fit well into an e-Learning*			
Written text	7.5/2.8	8.0/3.0	7.0/2.0
Spoken text (audio)	6.5/3.0	6.0/3.0	8.0/4.5
Graphics	9.0/2.0	9.0/2.0	9.0/2.0
Animated graphics	10.0/1.0	9.0 /1.0	10.0/0.5
Application videos	10.0/1.0	10.0/1.0	10.0/0.5
Animated explainer videos	9.5/1.0	10.0/1.0	9.0/2.0
Expert interviews (video)	7.0/2.8	6.0/3.0	8.0/1.5
Multiple choice questions	9.0/3.0	9.0/2.0	7.0/3.5
Assignments to be solved independently (to be uploaded)	7.5/2.8	8.0/3.0	7.0/2.5
Further reading (e.g., weblinks)	8.0/4.0	8.0/4.0	8.0/3.0
*Which communication channels would you expect to use during an e-Learning program?*			
Active use of discussion forums (own contributions)	2.5/1.0	2.0/1.0	5.0/6.5
Passive use of discussion forum (reading only)	7.0/3.8	7.0/3.0	7.0/6.0
No use of discussion forums	4.0/5.8	4.0/5.0	3.0/7.5
Possibility to contact the author via e-mail	3.5/7.0	3.0/4.0	9.0/8.5
Possibility to contact the responsible editor via e-mail	7.0/5.8	6.0/4.0	10.0/4.0
Possibility to contact other participants of my course	3.0/4.0	3.0/3.0	7.0/7.5

Numeric rating scale from 1 to 10 with 1 = strongly disagree to 10 = strongly agree. ^1^Students/head students affairs

^2^Oncology physicians

Overall, every proposed multimedia element was evaluated in a positive manner. Participants considered all of the suggested elements suitable for use in an e-Learning program. (Animated) graphics, videos, and application videos were preferred to written or spoken text (audio). The written text was preferred to spoken text (audio). Both multiple-choice questions and assignments to be solved independently were evaluated as suitable elements to check for knowledge gain.

Through comparing the different target groups (undergradute, postgraduate), there were no relevant differences in acceptance for the majority of the proposed elements, with one exception that expert interviews were perceived as more appropriate by postgraduates than by undergraduates.

Participants also reflected the communication channels to be used within an e-Learning program. Participants considered contact with the responsible editor or author to be more important than contact with other participants. In this survey, discussion forums were of minor importance. Participants considered the active use of discussion forums, i.e., posting of own contributions, as it is even less likely than reading the posts of others. Furthermore, interaction with other participants, experts, or authors seemed to be more important for postgraduates than for undergraduates.

### Phase 3: Importance of e-Learning Elements for Knowledge Gain

The importance of various elements for knowledge gain as evaluated by the participants after completing the e-Learning program is shown in Table 2. Multiple choice questions were perceived as particularly important. Likewise, written text and (animated) graphics were evaluated as particularly appropriate to gain knowledge. Application videos, further readings, and animation videos were also rated as important for learning. According to the evaluating participants, assignments to be solved independently and spoken text did not increase knowledge about the e-Learning program topics.

**Table 2:** Importance of e-Learning elements for knowledge gain (evaluation after e-Learning program)

	Total (*N* = 22) Number of mentions (% of total possible mentions)	Undergraduate^1 ^(*N* = 17) Number of mentions (% of total possible mentions)	Postgraduate^2 ^(*N* = 5) Number of mentions (% of total possible mentions)
Subjective knowledge gain: suitable elements			
Multiple choice questions	18 (81.8%)	14 (82.4%)	4 (80.0%)
Written text	15 (68.2%)	12 (70.6%)	3 (60.0%)
Animated graphics	15 (68.2%)	13 (76.5%)	2 (40.0%)
Graphics	13 (59.1%)	11 (64.7%)	2 (40.0%)
Application videos	12 (54.5%)	11 (64.7%)	1 (20.0%)
Animated explainer videos	12 (54.5%)	10 (58.8%)	2 (40.0%)
Expert interviews (video)	7 (31.8%)	6 (35.3%)	1 (20.0%)
Further reading (e.g., weblinks)	7 (31.8%)	6 (35.3%)	1 (20.0%)
Spoken text (audio)	4 (18.2%)	4 (23.5%)	0 (0%)
Assignments to be solved independently (to be uploaded)	2 (9.1%)	2 (11.8%)	0 (0%)

^1^Students/head students affairs

^2^Oncology physicians

### Phase 4: Knowledge Gain Assessed by a Progress Test

The increase in knowledge for students was determined by using a progress test (Table 3). The e-Learning program increased the number of single-choice questions answered correctly on the first attempt from an average of before 13.8 ± 2.3 to 21.2 ± 1.4 after, for a total of 23 questions. A considerable increase in knowledge was observed in all three training units. For the total score and the single training units, the increase in correctly answered questions was higher than one standard deviation. The validity of the questions was monitored by item analysis on the learning platform (Supplementary Material S5) [19].

**Table 3:** Students’ scores before and after e-Learning program

	Score before e-Learning (*N* = 15) mean ± standard deviation	Score after e-Learning (*N* = 15) mean ± standard deviation	Difference in score after-before e-Learning (*N* = 15) mean ± standard deviation
Total score (0–23)	13.8 ± 2.3	21.2 ± 1.4	7.4 ± 1.7
Chinese medicine (0–6)	4.1 ± 1.1	5.9 ± 0.3	1.8 ± 1.0
Mind body medicine (0–7)	4.6 ± 0.9	6.7 ± 0.5	2.1 ± 0.9
Whole medical systems (0–10)	5.1 ± 1.5	8.6 ± 1.2	3.5 ± 1.3

### Phase 5: Qualitative Interviews with Students: Focus Group Interview

In general, the students were very satisfied with the e-Learning program, the choice of topics and content, graphics, and didactic design of the lessons. It turned out that a majority of the students appreciated learning at their own pace, including self-assessment tests. Several students reported being more motivated to learn by answering single-choice-questions before and after the e-Learning program. The students appreciated the harmonization of their different knowledge levels by e-Learning so that on-site workshops could be built on comparable knowledge.

The short animated summaries at the end of each lesson were subjectively evaluated positively by the students, but they would see a printable summary as an advantage. Likewise, students desired an overview of the structure of the entire e-Learning program, indicating the required time before starting the course.

The majority of students stated that they remembered between 60 and 75% of the e-Learning content when they entered the on-site workshops.

### Phase 6: Integration of e-Learning Program in the KOKON-KTO Training for Oncology Physicians

For the KOKON-KTO training, an extended e-Learning program was used as preparation for a 2-day on-site skills training workshop [11]. As reported in [11], the e-Learning program was assessed positively (Supplementary Material S4).

## Discussion

In this study, we present a stepwise procedure involving experts and stakeholders to implement an optimized e-Learning program on complementary and integrative medicine. As shown here and in [2, 11], the e-Learning program led to a measurable knowledge gain in both target groups, postgraduates (oncology physicians) and undergraduates (medicine master’s students). Both tested target groups showed similar preferences concerning suitable e-Learning elements and were satisfied with the training. With a total of 5 to 9 learning units of 45 min each, the training is suitable for integration into everyday medical work but also allows deeper exploration of individual topics.

Our methodological approach has several strengths. We put emphasis on involving stakeholders from both target groups and an expert panel. We used a systematic stepwise approach that also follows the rules for developing medical curricula, according to Kern [20]. The stakeholders were involved in stepwise development to design a learner-centered e-Learning program.

There are also limitations. The test group size for student teaching was limited, and the group has been composed of students from the same university. In addition, we did not assess to what extent the digital competence of the participants influenced wishes and knowledge increased. Furthermore, a direct comparison with face-to-face training is missing; however, there are already numerous studies that show comparability of e-Learning courses and face-to-face trainings [5]. Even though knowledge gain has been shown, it would be desirable to repeat the progress test a longer time after e-Learning to also examine longer-term learning effects. It would also be interesting if the participants had similarly stated their knowledge gain in a self-assessment or if the knowledge gain would have been similar to a text-only e-Learning.

The evaluation of the e-Learning carried out up to this point reflects the first two stages of the 4-step evaluation model according to Kirkpatrick “reaction” and “learning” [21]. Level 1 (“reaction”) does not measure any increase in knowledge but rather the learners’ general satisfaction and motivation. Level 2 (“learning”), on the other hand, measures knowledge gain. The evaluation level 3 recommended by Kirkpatrick (“behavior”, i.e., application of knowledge in the actual context) and 4 (“results”, i.e., effects on patients) are not included in this project surveyed but are part of the analysis of the KOKON-KTO training and were analyzed in this context [2, 18].

An essential part of the conception of the e-Learning program was the definition of learning objectives and the selection of topics. These are based on the target group needs identified in previous projects [10] and the educational competencies for integrative oncology [22]. The learning objectives and topics focus on cognitive skills with clinical relevance, taking into account the multiple usabilities of the e-Learning program as stand-alone training or as part of blended learning concepts, which, e.g., convey information and communication strategies on this theoretical basis [18].

The process proposed here for creating the training content includes an e-Learning editor as a key role. This editor ensures the coordination of the authors, acts as a link between the expert panel and the authors, supports and advises authors in designing their learning content using its e-Learning expertise, and creates valid assessments. This role might also be crucial to connect e-Learning with on-site workshops in the case of blended learning scenarios.

As the participants perceived a variety of elements suitable for e-Learning programs, versatility in e-Learning seems to be advantageous compared to the exclusive use of, e.g., text or video. This is not surprising since different elements maintain learners’ engagement, attention, and satisfaction [23]. Learner engagements seem to remain rather constant from a student to professional since two target groups had the same preferences for elements. Our findings show only one exception: The opinion of experts played a rather important role for postgraduates but not for undergraduates. That might be explained by the fact that undergraduates’ needs are already satisfied by working out basic knowledge; postgraduates instead might want to go further than that and appreciate expert opinions as a substitute for professional exchange with colleagues. This is supported by our finding that interaction with other participants, experts, or authors is more important for postgraduates than undergraduates.

Interestingly, there was no pronounced need among both participant groups to use the learning platform for communication, e.g., in discussion forums. In particular, undergraduates would use forums even less than postgraduates would. Since discussion forums represent a rather work-intensive tool and their importance for the participants is, according to our data, low, no forum was developed and maintained in this project. The importance of exchange is likely to be higher for e-Learning programs, which convey not only cognitive skills but also aspects such as communication and application in clinical practice, where a clear need for feedback forums (with feedback from other health professionals, instructors, and patients) has been described [24].

During the implementation, the contribution of elements to learning success was examined. Particularly, both target groups valued the implementation of single choice questions into the training. In the e-Learning program described here, single choice questions are used as a formative assessment tool at the end of each lesson. The single choice questions are tailored to learning objectives and content. Participants could answer them repeatedly. After processing the questions, the solutions and explanations (explanatory feedback) for each question item are presented, which offers the opportunity for self-review and self-correction. This offers the learners certainty that they have achieved the required learning objectives and understood the learning content. Other studies also indicate that explanatory feedback increases student satisfaction [25]. Furthermore, it should be emphasized that the learners evaluated spoken texts (audios) as unfavorable by the learners for knowledge gain. Other studies that assessed learner satisfaction as a variable reported higher satisfaction among learners using audio [25].

The objective analysis of the increase in knowledge in students using a progress test is showing that the learning objectives of the e-Learning program are achieved. Learning success can be measured by a progress test [16] if learning objectives, learning content, and assessment questions are aligned according to the rules of constructive alignment as in this project [15]. The students had a high a priori knowledge of complementary medicine since an average of 56% of the questions were answered correctly before the e-Learning program. However, after e-Learning, this knowledge increased to an average of 91% of correctly answered questions, with an increase of more than one standard deviation. This result is consistent for the entire course and each of the three subareas examined. 

## Conclusion

The following recommendations for the further development of e-Learning courses can be derived from the data collected in the individual phases and the experience gained here. Before gathering content, the editorial process must be defined. A versatile selection of (multimedia) elements should be used if possible. Audio elements are less valuable in teaching than graphical representation and text. For the participants, the display of learning objectives as well as training structure and lesson length was helpful. Furthermore, participants appreciated formative assessment tools in the lessons and final tests in the complete course for self-control and motivation.

## Supplementary Information

Below is the link to the electronic supplementary material.

Supplementary file1 (PDF 80.1 KB)

Supplementary file2 (PDF 79.2 KB)

Supplementary file3 (PDF 84.3 KB)

Supplementary file4 (PDF 95.1 KB)

Supplementary file5 (PDF 139 KB)
