# Supplementary material for: Development, Implementation, and Evaluation of an e-Learning in Integrative Oncology for Physicians and Students Involving Experts and Learners: Experiences and Recommendations
Source: J Cancer Educ. 2022 Jul 1;38(3):805–12. doi: 10.1007/s13187-022-02189-1 (PMC9247929; doi:10.1007/s13187-022-02189-1)
Supplement: Supplementary file 1 — Supplementary file1 (PDF 80.1 KB) [file 13187_2022_2189_MOESM1_ESM.pdf]

Journal of Cancer Education

Development, implementation and evaluation of an e-Learning in integrative oncology for physicians and students involving experts and learners: Experiences and recommendations

Anita V. Thomae, Alizé A. Rogge, Stefanie M. Helmer, Katja Icke, Claudia M. Witt

Supplementary material 1: Characterization of the project phases

Phase 1:
Development of a concept for the e-Learning and content development
Discuss and define learning objectives, structure, authors

↓

Phase 2:
Assessing expectations and wishes for an e-Learning
Review and test of selected lessons, technical usability, design; N=20

↓

Phase 3:
Importance of e-Learning elements for knowledge gain
Implementation of further lessons including the knowledge gained in phase 2, quantitive assessment of subjective importance of e-Learning elements for knowledge gain; N=22

↓

Phase 4:
Knowledge gain assessed by a progress test
Implementation of the complete course, integration into a mastercourse medicine (blended learning), quantitiative assessment of knowledge gain; N=15

↓

Phase 5:
Qualitative interviews with medical students: Focus group interview
N=15

↓

Phase 6:
Integration of the e-Learning in the KOKON-KTO Training for oncology physicians
(blended learning); N=37
