# Supplementary material for: Development, Implementation, and Evaluation of an e-Learning in Integrative Oncology for Physicians and Students Involving Experts and Learners: Experiences and Recommendations
Source: J Cancer Educ. 2022 Jul 1;38(3):805–12. doi: 10.1007/s13187-022-02189-1 (PMC9247929; doi:10.1007/s13187-022-02189-1)
Supplement: Supplementary file 2 — Supplementary file2 (PDF 79.2 KB) [file 13187_2022_2189_MOESM2_ESM.pdf]

Journal of Cancer Education

Development, implementation and evaluation of an e-Learning in integrative oncology for physicians and students involving experts and learners: Experiences and recommendations

Anita V. Thomae, Alizé A. Rogge, Stefanie M. Helmer, Katja Icke, Claudia M. Witt

Supplementary material 2: Structure of the e-Learning course and learning objectives

- Module 1: Complementary and integrative medicine for cancer
- Module 2: Complementary and integrative medicine therapies
- Module 3: KOKON-KTO consultation

After the entire e-learning program, participants should:

- be able to classify the need and challenge of complementary medicine in cancer.
- be able to differentiate between various complementary medicine and other supportive procedures.
- be able to apply the essential elements of a KOKON conversation by example.

After “Module 1: Complementary and integrative medicine for cancer “ participants should be able:

- to name possible reasons for the use of complementary medicine and other supportive procedures in cancer patients.
- to name expectations of cancer patients in connection with complementary medicine.
- to classify how their attitude toward complementary medicine might influence their doctor–patient interaction.
- to name different provider groups.

After “Module 2: Complementary and integrative medicine therapies”, participants should be able:

- to name common complementary medicine and other supportive procedures.
- to explain the relevant characteristics of complementary medicine and other supportive procedures.
- to critically evaluate complementary medicine and other supportive procedures in cancer.

After “Module 3: KOKON-KTO consultation”, participants should be able:

- to state components of a KOKON-KTO conversation.
- to recognize elements of the conversation guide by example.
- to state challenges in conversations about complementary medicine.
- to make meaningful decisions in dealing with challenges in complementary medicine conversations.
