# Supplementary material for: Development, Implementation, and Evaluation of an e-Learning in Integrative Oncology for Physicians and Students Involving Experts and Learners: Experiences and Recommendations
Source: J Cancer Educ. 2022 Jul 1;38(3):805–12. doi: 10.1007/s13187-022-02189-1 (PMC9247929; doi:10.1007/s13187-022-02189-1)

Journal of Cancer Education

Development, implementation and evaluation of an e-Learning in integrative oncology for physicians and students involving experts and learners: Experiences and recommendations

Anita V. Thomae, Alizé A. Rogge, Stefanie M. Helmer, Katja Icke, Claudia M. Witt

Supplementary material 3: Stepwise development of online learning units

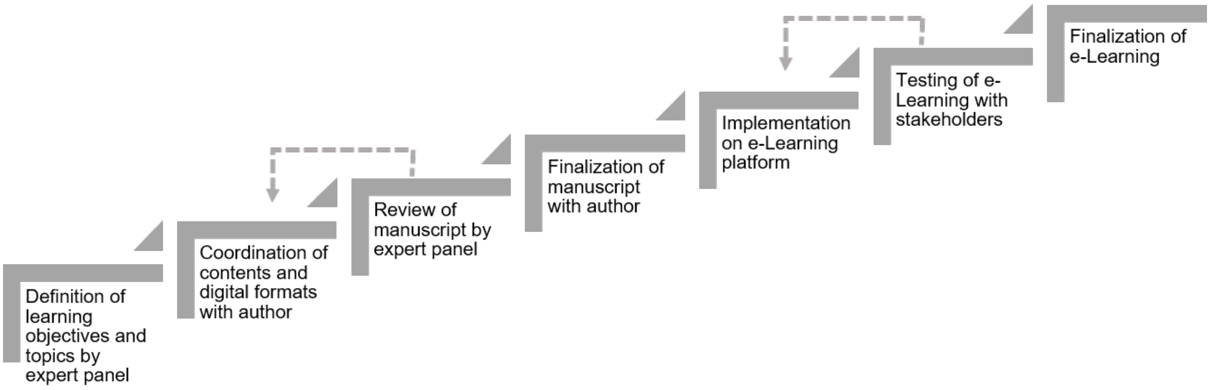

Supplement: Supplementary file 3 — Supplementary file3 (PDF 84.3 KB) [file 13187_2022_2189_MOESM3_ESM.pdf]
