# Supplementary material for: Development, Implementation, and Evaluation of an e-Learning in Integrative Oncology for Physicians and Students Involving Experts and Learners: Experiences and Recommendations
Source: J Cancer Educ. 2022 Jul 1;38(3):805–12. doi: 10.1007/s13187-022-02189-1 (PMC9247929; doi:10.1007/s13187-022-02189-1)
Supplement: Supplementary file 4 — Supplementary file4 (PDF 95.1 KB) [file 13187_2022_2189_MOESM4_ESM.pdf]

Journal of Cancer Education

Development, implementation and evaluation of an e-Learning in integrative oncology for physicians and students involving experts and learners: Experiences and recommendations

Anita V. Thomae, Alizé A. Rogge, Stefanie M. Helmer, Katja Icke, Claudia M. Witt

Supplementary material 4: Evaluation results e-Learning program for oncology physicians. Items were scored on a categorical scale ranging from 1 (strongly disagree) to 4 (strongly agree). (N=37)

	% Strongly disagree	% Disagree	% Agree	% Strongly agree
Interesting content	0	4.8	33.3	61.9
Subject-relevant content	4.8	9.5	38.1	47.6
Satisfaction from learning progress	4.8	14.3	52.4	28.6
Usefulness for working with cancer patients	0	14.3	38.1	42.9
Easy to understand training materials	0	0	57.1	42.9
Good e-learning in general	4.8	19	42.9	33.3

Results from Witt CM, Helmer SM, Schofield P, Wastell M, Canella C, Thomae AV, et al. Training oncology physicians to advise their patients on complementary and integrative medicine: An implementation study for a manual-guided consultation. Cancer. 2020;126(13):3031-41.
