# Supplementary material for: Development, Implementation, and Evaluation of an e-Learning in Integrative Oncology for Physicians and Students Involving Experts and Learners: Experiences and Recommendations
Source: J Cancer Educ. 2022 Jul 1;38(3):805–12. doi: 10.1007/s13187-022-02189-1 (PMC9247929; doi:10.1007/s13187-022-02189-1)
Supplement: Supplementary file 5 — Supplementary file5 (PDF 139 KB) [file 13187_2022_2189_MOESM5_ESM.pdf]

Journal of Cancer Education

Development, implementation and evaluation of an e-Learning in integrative oncology for physicians and students involving experts and learners: Experiences and recommendations

Anita V. Thomae, Alizé A. Rogge, Stefanie M. Helmer, Katja Icke, Claudia M. Witt

Supplementary material 5: Item analysis results for single-choice questions in progess test (N=15 attempts)

Figure 1: Facility index for single-choice question in progress test (first attempts). Data are shown for the initial test (before e-Learning program) and the final test (after completing the e-Learning program)

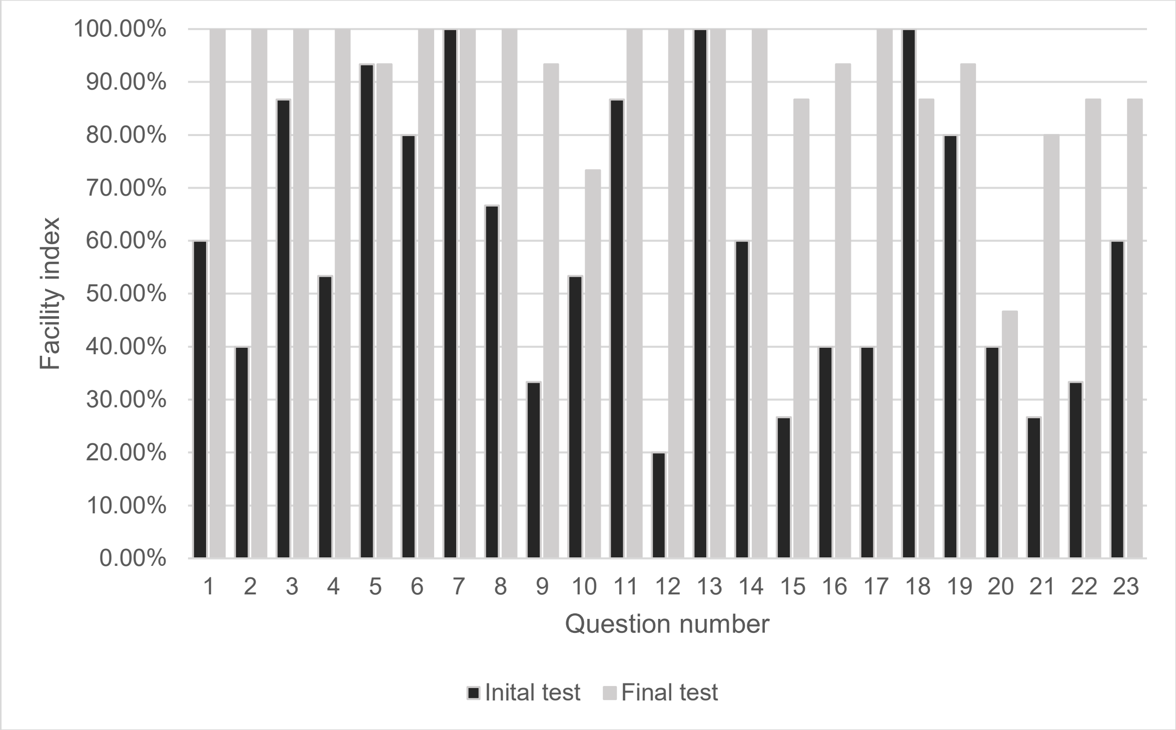

Single-choice questions

Please note: The rating "correct" or "incorrect" for individual items refers to the correct selection on the learning platform. In the case of negatively formulated questions (IS NOT), the answer marked as "correct" is the one that must be selected (i.e. the wrong item regarding the content). On the platform, items of each question are shown in arbitrary order.

Question #1

Which of the following **is not** one of the therapeutic methods of Chinese medicine?

A) Chinese nutrition therapy
B) Yoga
C) Acupuncture
D) Qi Gong
E) Tuina

Question #2

What is an important diagnostic method of Chinese medicine?

A) Tongue diagnosis
B) Hair mineral analysis
C) Urine inspection
D) Acid-base balance analysis
E) Vein diagnosis

Question #3

In the case of long-term use of Chinese drug therapy, the liver and kidney parameters in the blood must be checked regularly for safety reasons. Some patient groups are particularly at risk. Which of the following patient groups **does not** belong to this group?

A) Patients with known liver disease
B) Patients older than 65 years
C) Patients taking other medicines
D) Patients with functional gastrointestinal disorders
E) Patients for apply Chinese medicines at high doses

Question #4

What is moxibustion?

A) The stimulation of acupuncture points by palpation
B) The addition of burning herbs to acupuncture treatment
C) The application of acupuncture needles in the head area
D) The stimulation of acupuncture points with laser light
E) The use of cupping glasses instead of acupuncture needles

Question #5

What is the aim of acupuncture treatment in terms of Chinese medicine?

A) Generating a local counter-reaction to a stimulus
B) Desensitization of the nerve fibres in the skin
C) Harmonization of the life energy Qi
D) Draining out harmful fluids
E) Provoking a paradox counter-reaction

Question #6
The side effects of acupuncture are well studied. Which of the following side effects occurs frequently (i.e. 1 to 10 in 100 people treated)?

A) Minor bleeding at the injection site
B) Local infections
C) Heart palpitations
D) Vomiting
E) Injuries to nerves

Question #7
What **is not** an important core element of a Mindfulness-Based Stress Reduction (MBSR) course?

A) Mindful body awareness
B) Mindful body exercises
C) Sitting meditation
D) Conversion to a vegetarian diet
E) Daily exercise sessions for at home

Question #8
To which patients should mindfulness-based practices only be recommended with caution?

A) Tumor patients
B) Chronic pain patients
C) Schizophrenia patients
D) Depressed patients
E) Patients with irritable bowel syndrome

Question #9
What evidence is known for the effectiveness of autogenic training in cancer patients?

A) There is evidence that sleep patterns could be improved.
B) There is evidence that menopausal symptoms may be improved.
C) There is evidence that existing nausea may be improved.
D) There is evidence that appetite could be increased.
E) There is evidence that mucositis may improve.

Question #10
In which case should caution be taken when applying autogenic training?

A) For elder patients
B) For patients with overweight
C) For patients with active psychoses
D) For patients with a depression
E) For patients with severe mobility impairments

Question #11
What does Guided Imagination usually focus on?

A) On conscious body awareness
B) On pleasant thoughts
C) On the perception of the sound
D) On pain sensations
E) On the perception of muscle tension

Question #12
What **is not** a basic assumption of progressive muscle relaxation (PMR)?

A) Anxiety is accompanied by muscle tension.
B) Muscle relaxation occurs more easily after conscious muscle tension.
C) The targeted tensing of the muscles increases the ability to concentrate.
D) When muscles are relaxed, anxiety cannot be felt at the same time.
E) Muscle tension is increased in stressful situations.

Question #13
What is the aim of progressive muscle relaxation (PMR)?

A) The induction of relaxation
B) The induction of a light trance state
C) The induction of sleep
D) The induction of euphoria
E) The induction of reduced appetite

Question #14
Which of the following naturopathic procedures is considered a purgative procedure?

A) Hydrotherapy
B) Order therapy
C) Leech therapy
D) Exercise therapy
E) Nutrition therapy

Question #15
Which of the following applications **is not** a hydrotherapeutic measure?

A) Sauna
B) Alternating warm foot baths
C) Cold calf wraps
D) Kneipp water treading
E) Hydrocolon therapy

Question #16
Which of the following statements about naturopathic cupping is **false?**

A) Cupping can be done on intact skin.
B) With cupping, an artificial wound can be placed in the skin.
C) Cupping can be performed as massage.
D) Cupping is used to relieve pain in the musculoskeletal system.
E) With cupping, there is a risk of numerous side effects even when performed correctly.

Question #17
What **is not** a principle of anthroposophical therapy?

A) The therapy concept is individualized.
B) The therapy concept is multimodal.
C) The therapy is intended to support the self-regulating capacities
D) The therapy rejects conventional oncological treatment.
E) The therapy tries to achieve a harmonic interaction of the functional systems.

Question #18
In anthroposophic medicine, an individualised, multimodal therapy concept is created for the treatment of cancer patients.
What **is not** a typical component of such a therapy concept?

A) Biography work
B) Art therapy
C) Eurythmy therapy
D) Intake of amygdalin/laetrile
E) Wraps and compresses

Question #19
What is meant by the term "potentisation" in the homeopathic sense?

A) The gradual dilution and shaking of homeopathic medicines
B) The recording of symptoms caused by homeopathic medicines in a healthy person
C) The repeated administration of a homeopathic remedy
D) Creating a symptom profile of the patient
E) Enhancing the effect of the medicine through an individual discussion with the therapist

Question #20
The homeopathic remedy picture is an important element of homeopathic treatment. It is determined during the homeopathic drug trial and clinical application. Which of the following elements **does not** include a homeopathic drug trial?

A) The defined administration of the homeopathic remedy to healthy persons
B) Integration of toxicological findings
C) Recording of clinical experience
D) Effect in patients
E) Preparation of the homeopathic medicinal product

Question #21
What does the term "Pancakarma" mean?

A) "Pancakarma" refers to Ayurvedic meditation exercises.
B) "Pancakarma" refers to the internal purgative procedures of Ayurvedic medicine.
C) "Pancakarma" refers to the totality of Ayurvedic therapeutic measures.
D) "Pancakarma" refers to the vegetarian change of diet according to Ayurvedic principles.
E) "Pancakarma" refers to the external application of oils in Ayurvedic medicine.

Question #22
There are certain risks to be considered when using Ayurvedic medicines and procedures. Which statement about possible risks is **wrong**?

A) There is a potential for interaction between Ayurvedic and other medicines.
B) If not done properly, elements of Pancakarma can lead to fluid loss.
C) Ayurvedic preparations can be contaminated with heavy metals.
D) Complications from Pancakarma are possible in cases of pronounced organ damage.
E) Ayurvedic therapeutic measures should not be used for psychosomatic diseases.

Question #23
Which **is not** an essential element of Ayurvedic medicine?

A) Ayurvedic therapy measures are adapted to the constitution of the patients.
B) The doshas are always equally pronounced in healthy people.
C) Preventive measures are an important part of Ayurveda.
D) Meditation exercises are used in Ayurveda as a balancing procedure.
E) Draining procedures are designed to remove harmful substances from the body.
